# Supplementary material for: Pancreatic α and β cells are globally phase-locked
Source: Nat Commun. 2022 Jun 28;13:3721. doi: 10.1038/s41467-022-31373-6 (PMC9240067; doi:10.1038/s41467-022-31373-6)
Supplement: Supplementary file 1 — Supplementary Information [file 41467_2022_31373_MOESM1_ESM.pdf]

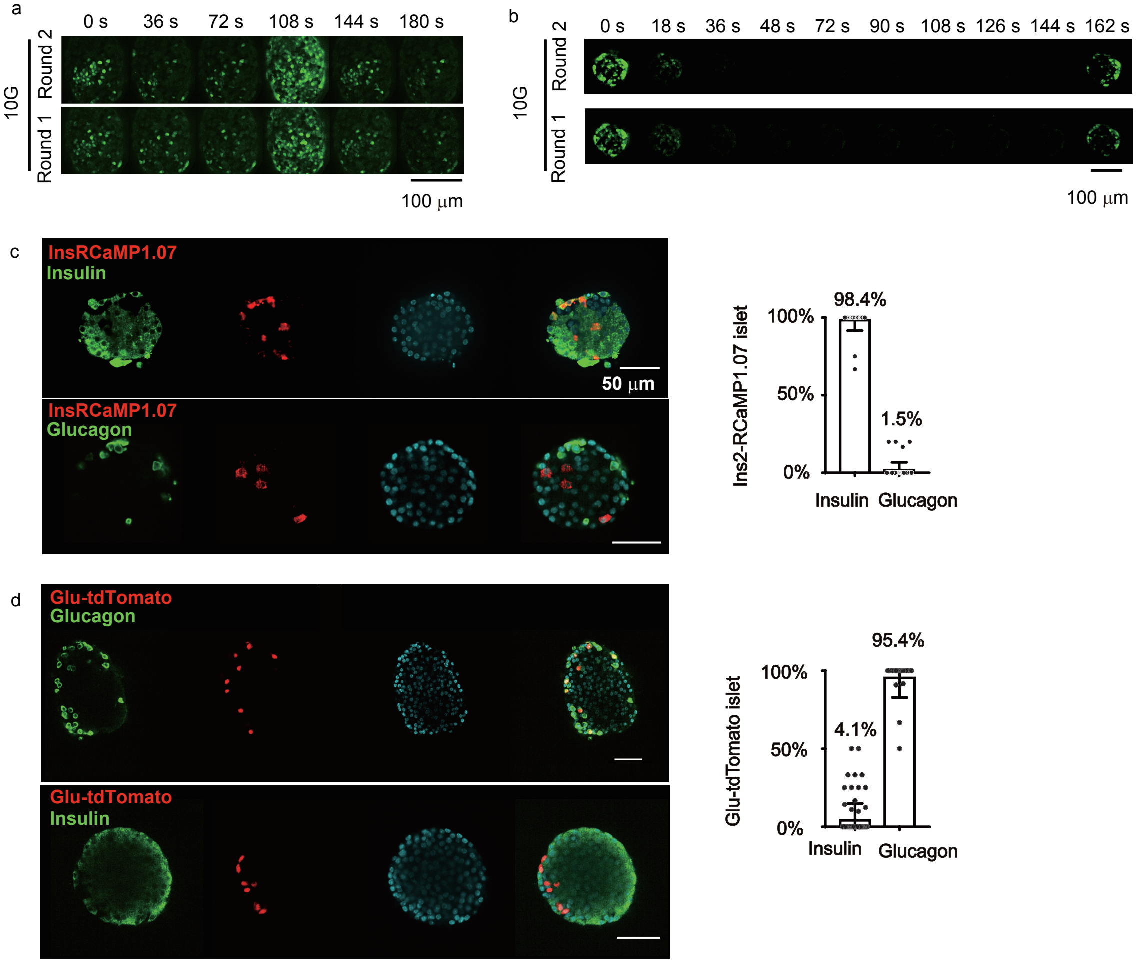
a
0 s
36 s
72 s
108 s
144 s
180 s
10G
Round 2
Round 1
100 µm
b
0 s
18 s
36 s
48 s
72 s
90 s
108 s
126 s
144 s
162 s
10G
Round 2
Round 1
100 µm
c
InsRCaMP1.07
Insulin
50 µm
InsRCaMP1.07
Glucagon
Ins2-RCaMP1.07 islet
98.4%
1.5%
100%
50%
0%
Insulin
Glucagon
d
Glu-tdTomato
Glucagon
Glu-tdTomato
Insulin
Glu-tdTomato islet
95.4%
4.1%
100%
50%
0%
Insulin
Glucagon

**Supplementary Fig. 1. RCaMP1.07 Expressing Islet Cells from Ins2-RCaMP1.07 Mice Are Insulin Positive, GCaMP6f Cells Glucagon Positive**

a) Cell activation sequence in the first and second round of 10G stimulation. We subtract the previous frame from the next frame of the original $Ca^{2+}$ images (frame interval 3 s). Shown is the maximal intensity projection in a mixed oscillatory islet (time interval was 36 s).
b) Same as (a) for a slow oscillatory islet (time interval was 30 s)
c) Confocal images of insulin (top) and glucagon (bottom) immunostained *Ins2-RCaMP1.07* islet. RCaMP1.07 expressing cells were 98.4% insulin positive (n=37 islets) and 1.5% glucagon positive (n=51 islets). Scale bars, 50 μm. Bars represent mean ± s.d..
d) Confocal images of glucagon (top) and insulin (bottom) immunostained *Glu-Cre*$^{+}$*;GCaMP6f*$^{f/+}$ islet. GCaMP6f expressing cells were 95.4% glucagon positive (n=22 islets) and 4.1% insulin positive (n=88 islets). Scale bars, 50 μm. Bars represent mean ± s.d..

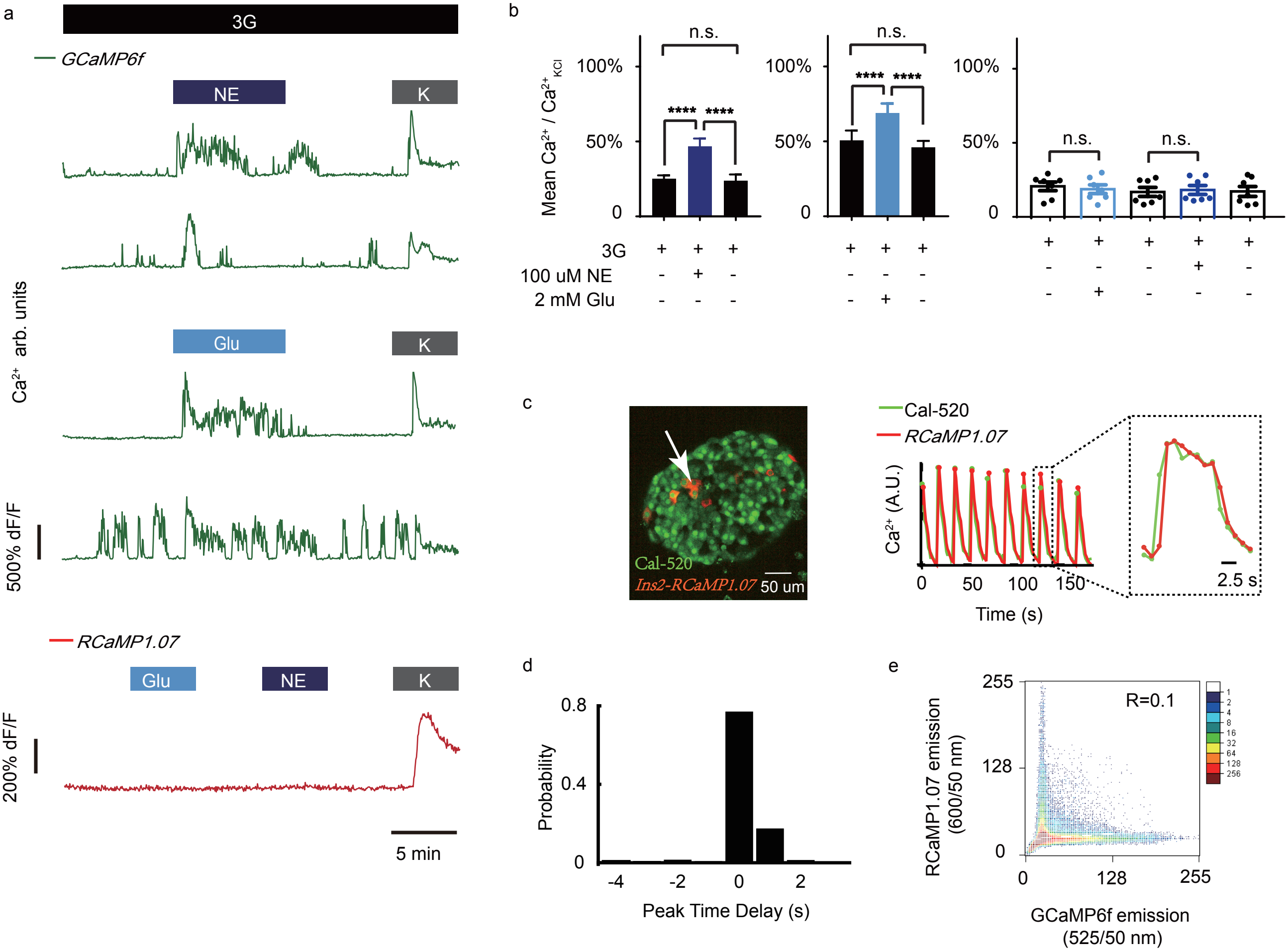

a
3G
GCaMP6f
NE
K
Glu
K
Ca2+ arb. units
500% dF/F
RCaMP1.07
Glu
NE
K
200% dF/F
5 min
b
Mean Ca2+ / Ca2+ KCl
n.s.
****
****
100%
50%
0
3G
100 uM NE
2 mM Glu
c
Cal-520
Ins2-RCaMP1.07
50 um
Cal-520
RCaMP1.07
Ca2+ (A.U.)
Time (s)
2.5 s
d
Probability
Peak Time Delay (s)
e
R=0.1
RCaMP1.07 emission (600/50 nm)
GCaMP6f emission (525/50 nm)

**Supplementary Fig. 2. GCaMP6f and RCaMP1.07 Emission Reflects $Ca^{2+}$ Dynamics in α and β Cells Accurately**

a) Top: Single cell $Ca^{2+}$ signal from intact *Glu-Cre⁺;GCaMP6f^f/+* islets exposed to 100 uM NE, 2 mM glutamate and 25 mM KCl stimulation (with 3 mM glucose). Bottom: Single cell $Ca^{2+}$ signal from intact *Ins2-RCaMP1.07* islet cells exposed to 100 uM NE, 2 mM glutamate and 25 mM KCl stimulation (with 3 mM glucose).

b) Mean $Ca^{2+}$ intensity (normalized to the $Ca^{2+}$ intensity with KCl stimulation) with and without NE (left, n=55 cells), glutamate (middle, n=63 cells) in *Glu-Cre⁺;GCaMP6f^f/+* islet cells. Right: Mean $Ca^{2+}$ intensity (normalized to the $Ca^{2+}$ intensity with KCl stimulation) with and without NE, glutamate in *Ins2-RCaMP1.07* islet cells, n = 7 cells. Bars represent mean ± s.d. Statistical comparisons are conducted using two way ANOVA test

c) Left: Maximal projection of $Ca^{2+}$ signal from intact *Ins2-RCaMP1.07* mouse islet loading green calcium indicator Cal-520 AM (10G). Right: Normalized β cell RCaMP1.07 emission (600nm*,* Red) and Cal-520 AM emission (525 nm, Green), the arrow in left panel shows the cell position. A pair of enlarged traces were shown.

d) Histogram showed the time delay between RCaMP1.07 and Cal-520 AM peaks. On average, the RCaMP1.07 peaks were 0.3 s earlier than Cal-520 peaks (n=12 β cells, the mean period was 19s, total duration was 8 min, time resolution was 1.3s). It suggested RCaMP1.07 worked as a sensitive calcium sensor.

e) The average correlation coefficient between the GCaMP6f emission (single band pass filter with center wavelength 525 nm, band width 50 nm) and RCaMP1.07 emission (single band pass filter with center wavelength 600 nm, band width 50 nm) is 0.19 (n=21 islets from 5 mice in 5 independent isolation).

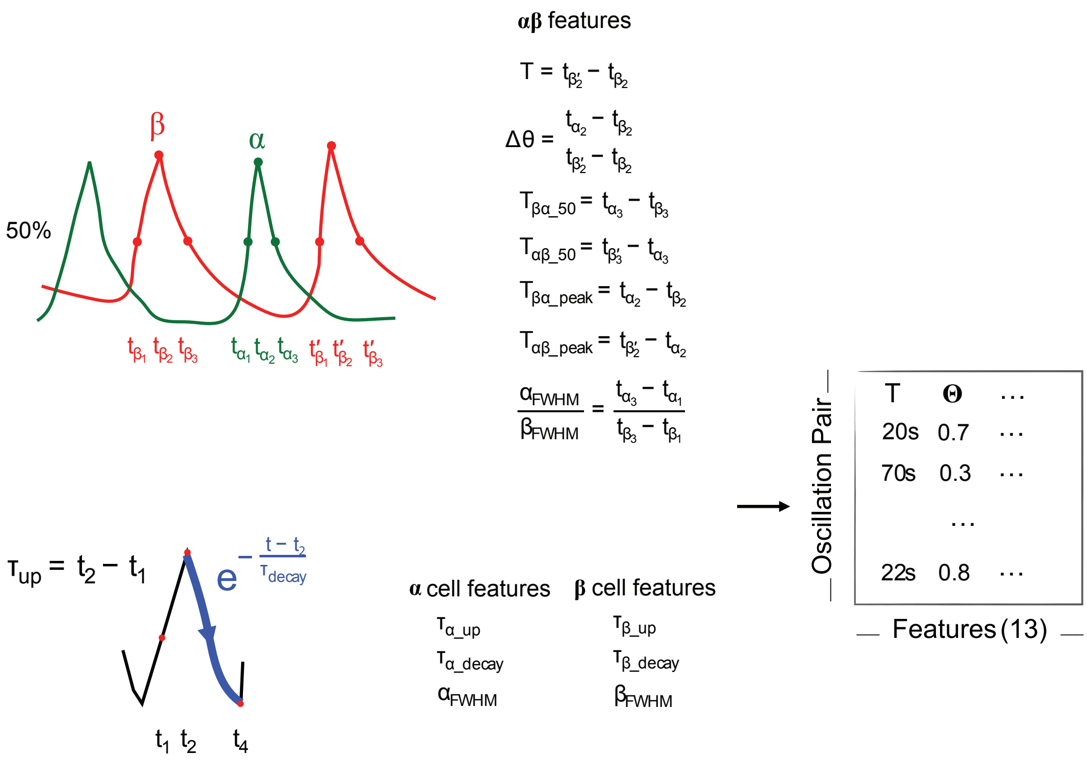

αβ features
T = t_β′2 − t_β2
Δθ = (t_α2 − t_β2) / (t_β′2 − t_β2)
T_βα_50 = t_α3 − t_β3
T_αβ_50 = t_β′3 − t_α3
T_βα_peak = t_α2 − t_β2
T_αβ_peak = t_β′2 − t_α2
α_FWHM / β_FWHM = (t_α3 − t_α1) / (t_β3 − t_β1)
β
α
50%
t_β1 t_β2 t_β3 t_α1 t_α2 t_α3 t′_β1 t′_β2 t′_β3
τ_up = t_2 − t_1
e^−(t − t_2)/τ_decay
t_1 t_2 t_4
α cell features
τ_α_up
τ_α_decay
α_FWHM
β cell features
τ_β_up
τ_β_decay
β_FWHM
Oscillation Pair
T Θ …
20s 0.7 …
70s 0.3 …
…
22s 0.8 …
Features (13)

**Supplementary Fig. 3. Definition of Features in α-β Oscillation Pair**

For each oscillatory pair, 13 features are defined, including 3 α features, 3 β features, and 7 α-β pair features. The definitions of α-β features are shown in the top panel. The definitions of α(β) features are shown in the lower panel, in which $T_{decay}$ is the decay time constant (fitted from an exponential function) from the peak to valley and FWHM is defined in Fig. 4a.

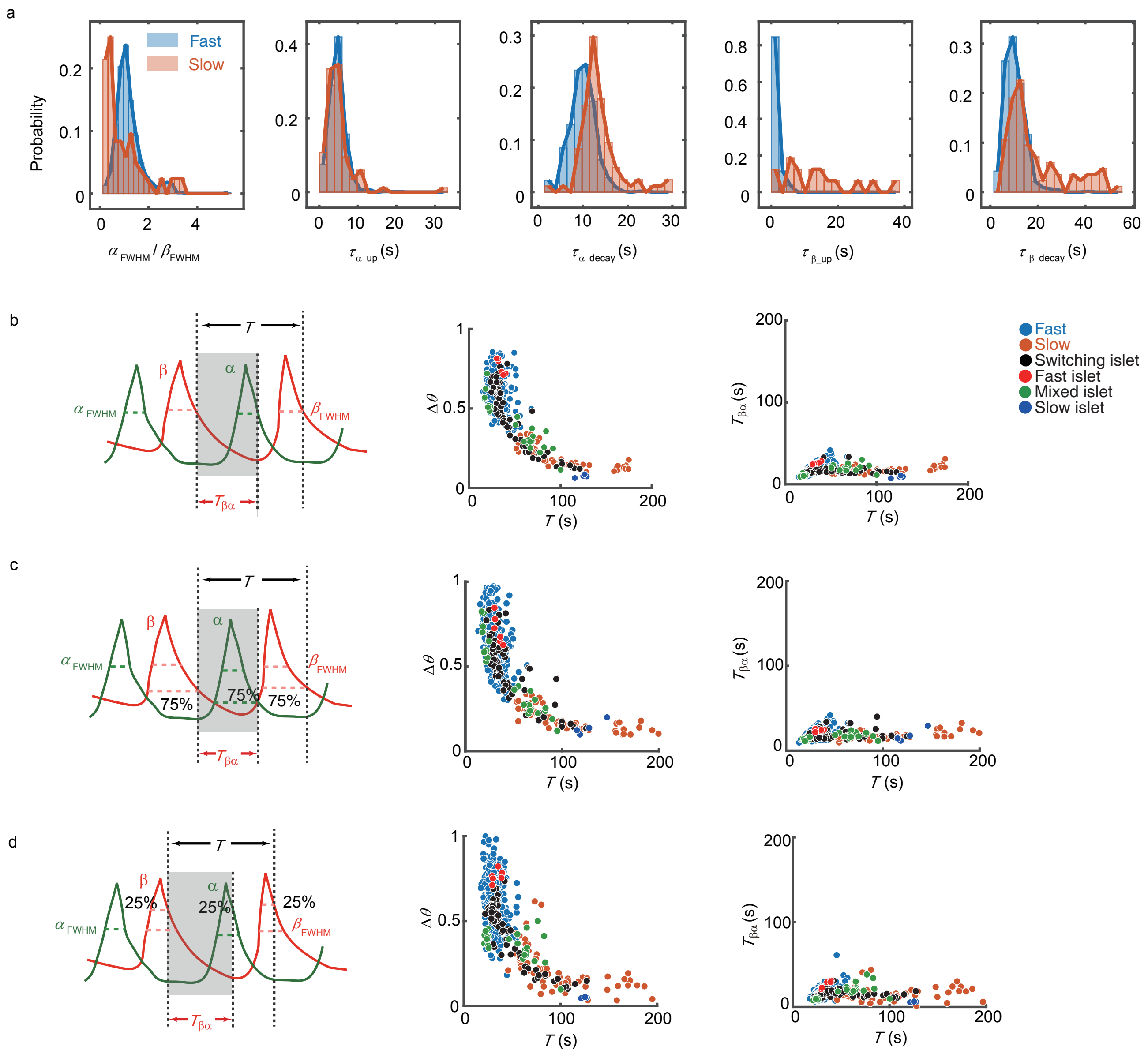

a
Probability
Fast
Slow
$\alpha_{FWHM} / \beta_{FWHM}$
$\tau_{\alpha\_up}$ (s)
$\tau_{\alpha\_decay}$ (s)
$\tau_{\beta\_up}$ (s)
$\tau_{\beta\_decay}$ (s)
b
T
β
α
$\alpha_{FWHM}$
$\beta_{FWHM}$
$T_{\beta\alpha}$
Δθ
T (s)
$T_{\beta\alpha}$ (s)
Fast
Slow
Switching islet
Fast islet
Mixed islet
Slow islet
c
75%
d
25%

**Supplementary Fig. 4. Results for Different Definitions of Periods and Waiting Times**

a) The various features compared between the fast (blue) and slow (orange) clusters in Fig. 4b.

b) Using time window at the 50% decrease of α and β cells' activation to define $T$ and $T_{\alpha\beta}$. Right: Same scatter plots as in Fig. 4d. Note the similar patterns between $T$ and $T_{\alpha\beta}$ with Fig. 4d.

c) Using time window at the 75% decrease of α and β cells' activation to define $T$ and $T_{\alpha\beta}$.

d) Using time window at the 25% decrease of α and β cells' activation to define $T$ and $T_{\alpha\beta}$.

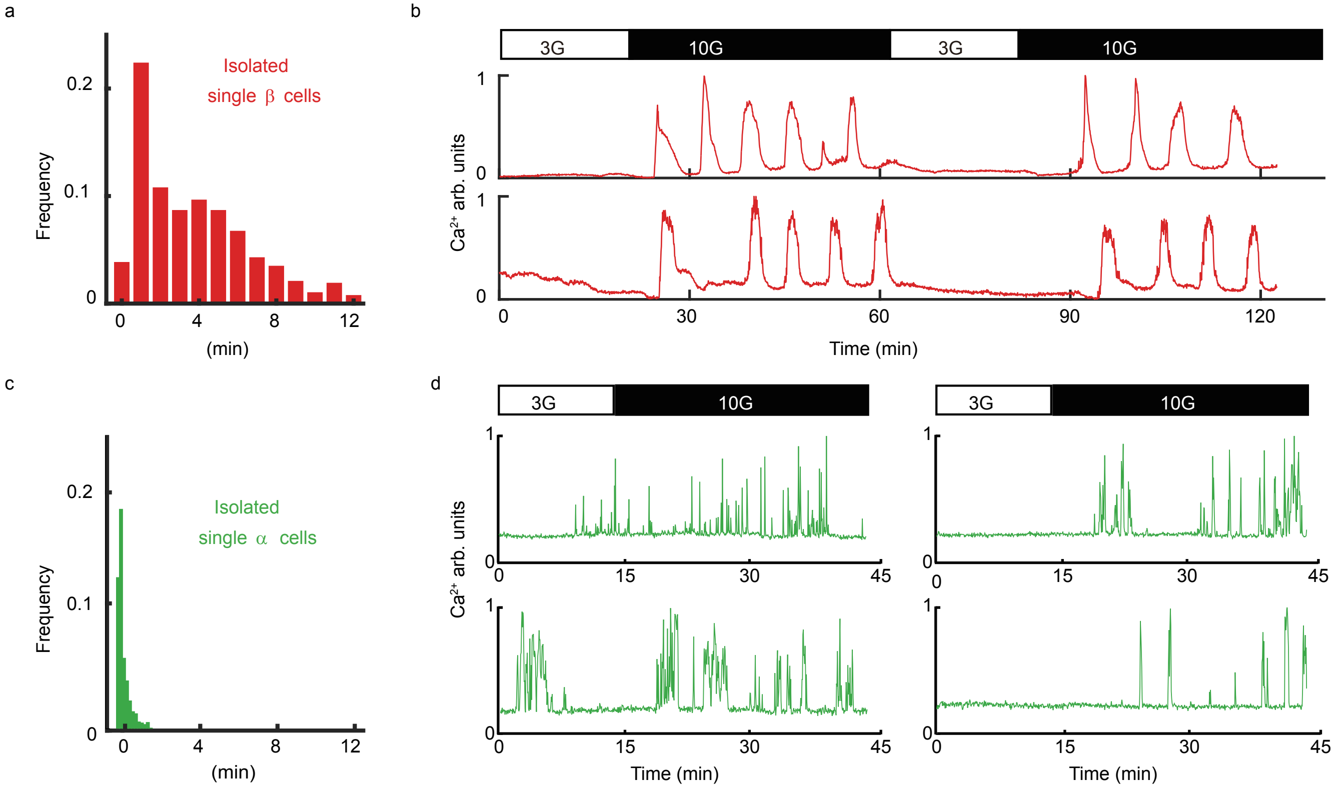

a
Frequency
0.2
0.1
0
0
4
8
12
(min)
Isolated
single β cells
b
3G
10G
3G
10G
Ca2+ arb. units
1
0
1
0
0
30
60
90
120
Time (min)
c
Frequency
0.2
0.1
0
0
4
8
12
(min)
Isolated
single α cells
d
3G
10G
3G
10G
Ca2+ arb. units
1
0
0
15
30
45
Time (min)

**Supplementary Fig. 5. Isolated Single α Cells Show Faster Oscillation than Isolated Single β Cells**

a) Distribution of oscillation period from the isolated free single β cells. Mean period is 4.9 min (n = 91 cells).

b) Typical $Ca^{2+}$ traces of free single β cells under repetitive 3G and 10G stimulation.

c) Distribution of oscillation period from the isolated free single α cells. Mean period is 43 s (n = 76 cells)

d) α cell under 3G and 10G stimulations. Left panel: 3G active and 10G active α cells (29 of 80 cells). Right panel: 3G inactive and 10G active α cells (14 of 80 cells).

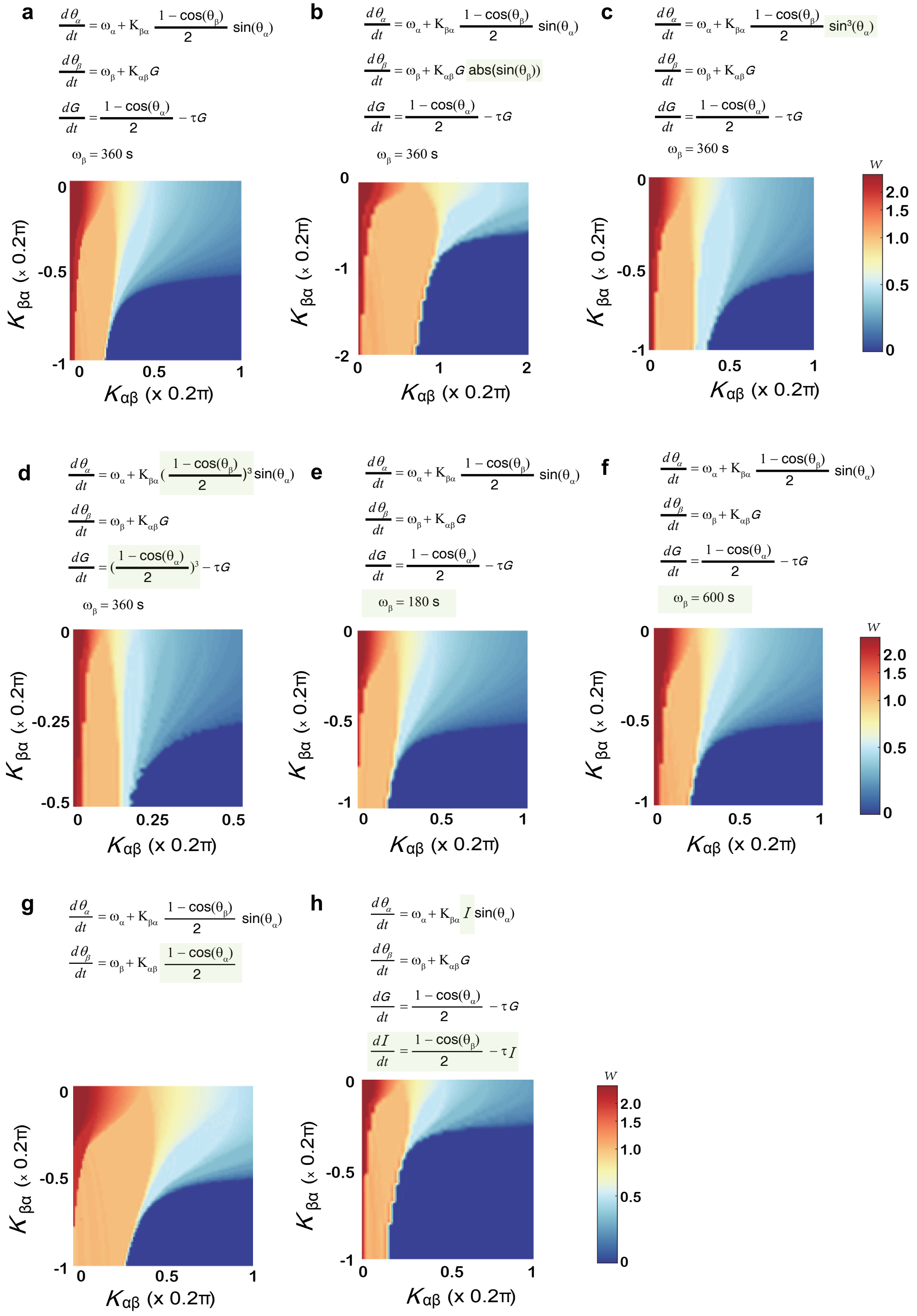

a
$\frac{d\theta_\alpha}{dt} = \omega_\alpha + K_{\beta\alpha}\frac{1-\cos(\theta_\beta)}{2}\sin(\theta_\alpha)$
$\frac{d\theta_\beta}{dt} = \omega_\beta + K_{\alpha\beta}G$
$\frac{dG}{dt} = \frac{1-\cos(\theta_\alpha)}{2} - \tau G$
$\omega_\beta = 360$ s
b
$\frac{d\theta_\alpha}{dt} = \omega_\alpha + K_{\beta\alpha}\frac{1-\cos(\theta_\beta)}{2}\sin(\theta_\alpha)$
$\frac{d\theta_\beta}{dt} = \omega_\beta + K_{\alpha\beta}G\,\mathrm{abs}(\sin(\theta_\beta))$
$\frac{dG}{dt} = \frac{1-\cos(\theta_\alpha)}{2} - \tau G$
$\omega_\beta = 360$ s
c
$\frac{d\theta_\alpha}{dt} = \omega_\alpha + K_{\beta\alpha}\frac{1-\cos(\theta_\beta)}{2}\sin^3(\theta_\alpha)$
$\frac{d\theta_\beta}{dt} = \omega_\beta + K_{\alpha\beta}G$
$\frac{dG}{dt} = \frac{1-\cos(\theta_\alpha)}{2} - \tau G$
$\omega_\beta = 360$ s
d
$\frac{d\theta_\alpha}{dt} = \omega_\alpha + K_{\beta\alpha}\left(\frac{1-\cos(\theta_\beta)}{2}\right)^3\sin(\theta_\alpha)$
$\frac{d\theta_\beta}{dt} = \omega_\beta + K_{\alpha\beta}G$
$\frac{dG}{dt} = \left(\frac{1-\cos(\theta_\alpha)}{2}\right)^3 - \tau G$
$\omega_\beta = 360$ s
e
$\frac{d\theta_\alpha}{dt} = \omega_\alpha + K_{\beta\alpha}\frac{1-\cos(\theta_\beta)}{2}\sin(\theta_\alpha)$
$\frac{d\theta_\beta}{dt} = \omega_\beta + K_{\alpha\beta}G$
$\frac{dG}{dt} = \frac{1-\cos(\theta_\alpha)}{2} - \tau G$
$\omega_\beta = 180$ s
f
$\frac{d\theta_\alpha}{dt} = \omega_\alpha + K_{\beta\alpha}\frac{1-\cos(\theta_\beta)}{2}\sin(\theta_\alpha)$
$\frac{d\theta_\beta}{dt} = \omega_\beta + K_{\alpha\beta}G$
$\frac{dG}{dt} = \frac{1-\cos(\theta_\alpha)}{2} - \tau G$
$\omega_\beta = 600$ s
g
$\frac{d\theta_\alpha}{dt} = \omega_\alpha + K_{\beta\alpha}\frac{1-\cos(\theta_\beta)}{2}\sin(\theta_\alpha)$
$\frac{d\theta_\beta}{dt} = \omega_\beta + K_{\alpha\beta}\frac{1-\cos(\theta_\alpha)}{2}$
h
$\frac{d\theta_\alpha}{dt} = \omega_\alpha + K_{\beta\alpha}I\sin(\theta_\alpha)$
$\frac{d\theta_\beta}{dt} = \omega_\beta + K_{\alpha\beta}G$
$\frac{dG}{dt} = \frac{1-\cos(\theta_\alpha)}{2} - \tau G$
$\frac{dI}{dt} = \frac{1-\cos(\theta_\beta)}{2} - \tau I$
$K_{\beta\alpha}$ (x 0.2π)
$K_{\alpha\beta}$ (x 0.2π)
W
2.0
1.5
1.0
0.5
0

**Supplementary Fig. 6. The Structure of the Phase Diagram Is Robust to the Functional Forms of Paracrine Interactions**

a) The original model. The color bar codes the winding number.

b) Different β cell hormone response function $f_{r\beta}(\theta)$.

c) Different α cell hormone response function $f_{r\alpha}(\theta)$.

d) Different hormone secretion function $f_{s}(\theta)$.

e) Faster β cell intrinsic oscillation period. Green shadow indicates the revised item.

f) Slower β cell intrinsic oscillation period. Green shadow indicates the revised item.

g) The interaction between the α cell and the β cell was instantaneous hypothesis based model. Green shadow indicates the revised item.

h) The interaction between the α cell and the β cell was determined by the accumulated level of glucagon and insulin hypothesis-based model. Green shadow indicates the revised item.

a

$$\frac{d\theta_\beta}{dt} = \omega_\beta + K_{\alpha\beta}\, f_s(\, f_t(\theta_\alpha))\qquad \frac{d\theta_\alpha}{dt} = \omega_\alpha - K_{\beta\alpha}\, f_s(\, f_t(\theta_\beta))\, f_{r\alpha}(\, f_t(\theta_\alpha))$$

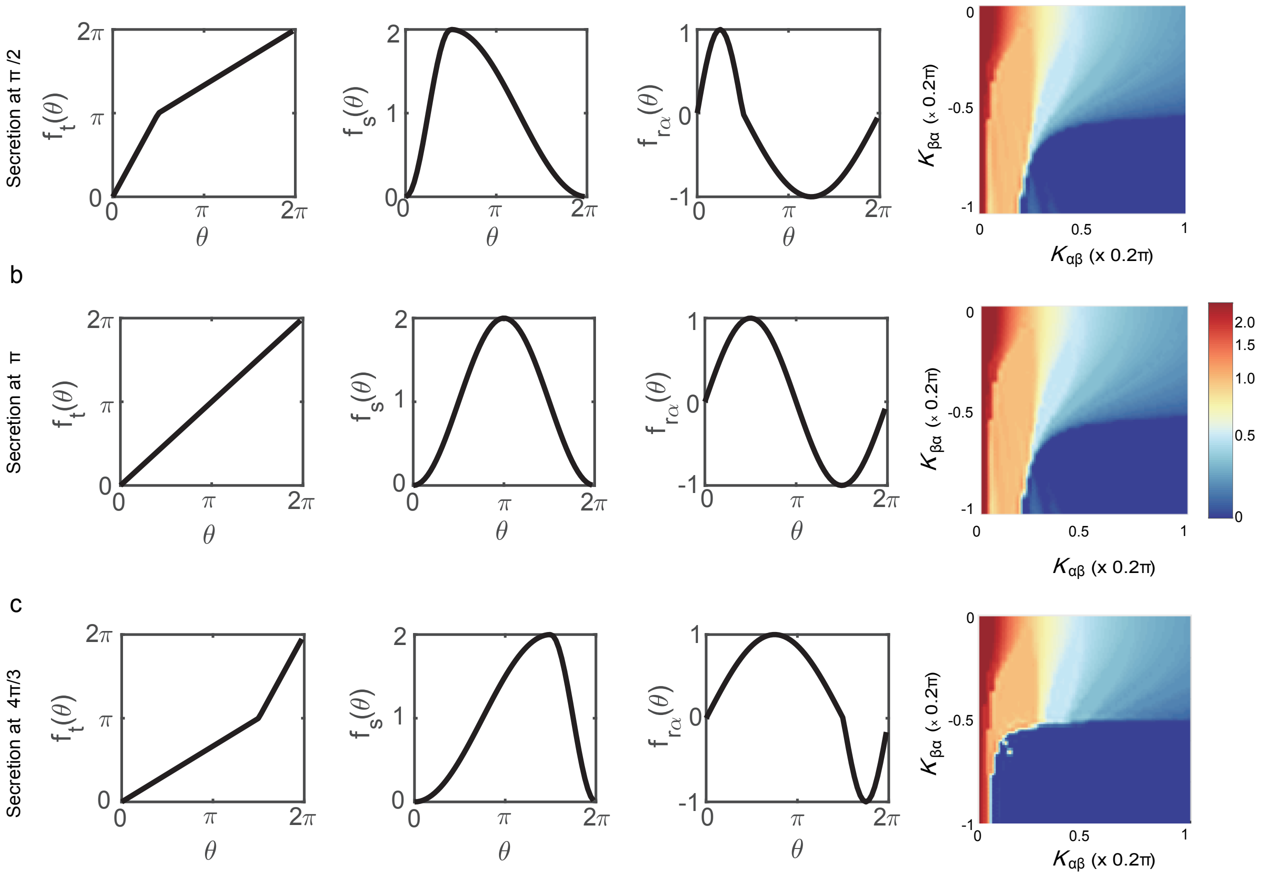

**Supplementary Fig. 7. The Structure of the Phase Diagram Is Robust to the Phase of Hormone Secretion**

a) Maximal secretion at phase $\pi/2$.

b) Maximal secretion at phase $\pi$ (original model).

c) Maximal secretion at phase $3\pi/4$.

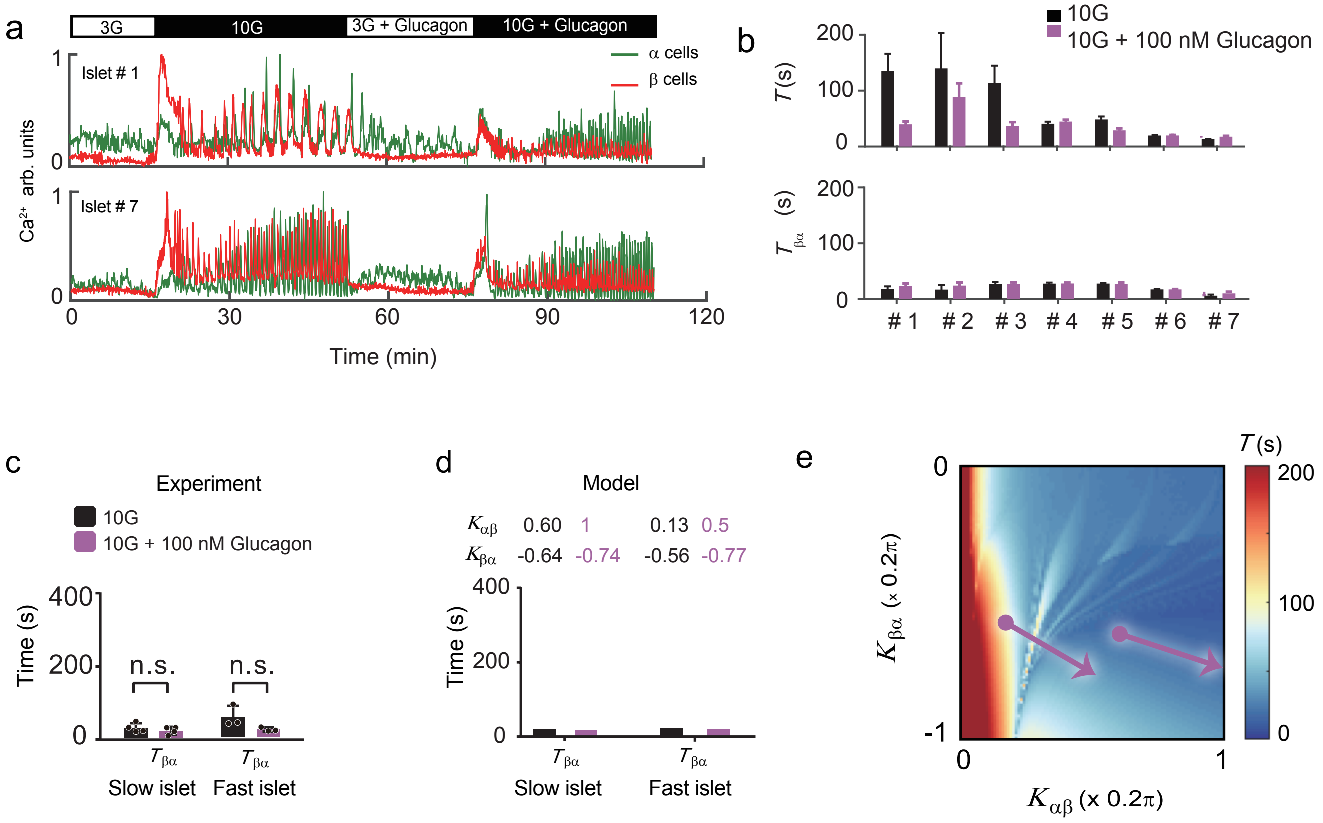

a
3G
10G
3G + Glucagon
10G + Glucagon
Islet # 1
Islet # 7
α cells
β cells
Ca2+ arb. units
Time (min)
b
10G
10G + 100 nM Glucagon
T (s)
$T_{\beta\alpha}$ (s)
# 1
# 2
# 3
# 4
# 5
# 6
# 7
c
Experiment
10G
10G + 100 nM Glucagon
Time (s)
n.s.
n.s.
$T_{\beta\alpha}$
Slow islet
Fast islet
d
Model
$K_{\alpha\beta}$ 0.60 1 0.13 0.5
$K_{\beta\alpha}$ -0.64 -0.74 -0.56 -0.77
Time (s)
$T_{\beta\alpha}$
Slow islet
Fast islet
e
T (s)
$K_{\beta\alpha}$ (x 0.2π)
$K_{\alpha\beta}$ (x 0.2π)

**Supplementary Fig. 8. Glucagon Speeds up Oscillation of Slow Islets but not Fast Islets**

a) Representative recordings of $Ca^{2+}$ in α and β cells in *Glu-Cre$^{+}$; GCaMP6f$^{f/+}$; Ins2-RCaMP1.07* mice islets, with consecutive stimulations of 3G (10 min), 10G (40 min), 3G+100nM glucagon (20 min) and 10G+100nM glucagon (40 min). Islets #1 and #7 are shown. Green for α and red for β cell.

b) Oscillation period ($T$) and waiting for the α cell activation following β cell activation ($T_{\beta\alpha}$) in each islet, with and without glucagon treatment (n=7 islets from 3 mice in 3 independent isolation, each islet has 3-30 oscillations, see Source Data). Bars represent mean ± s.d.

c) $T_{\beta\alpha}$ and $T_{\alpha\beta}$ from experiments under 10G stimulation with and without glucagon treatment (n = 3 slow oscillation islets and 4 fast oscillation islets from 3 mice in 3 independent isolation). Bars represent mean ± s.d.

d) $T_{\beta\alpha}$ and $T_{\alpha\beta}$ from the model in Fig. 7b top two rows.

e) Schematic of model perturbation for increasing glucagon in the phase diagram.

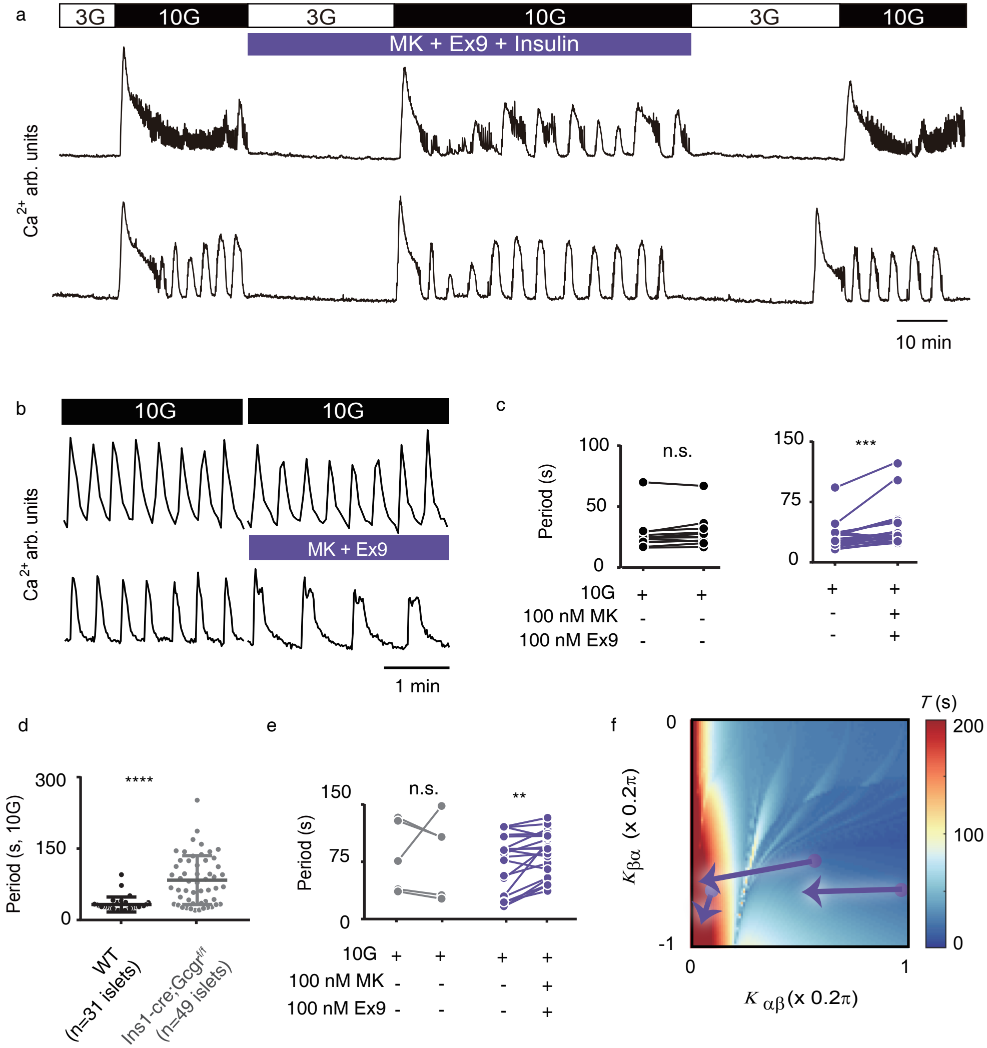
a
3G
10G
3G
10G
3G
10G
MK + Ex9 + Insulin
Ca2+ arb. units
10 min
b
10G
10G
MK + Ex9
Ca2+ arb. units
1 min
c
Period (s)
100
50
0
n.s.
150
75
0
***
10G
100 nM MK
100 nM Ex9
d
Period (s, 10G)
300
150
0
****
WT (n=31 islets)
Ins1-cre;Gcgr f/f (n=49 islets)
e
Period (s)
150
75
0
n.s.
**
10G
100 nM MK
100 nM Ex9
f
T (s)
200
100
0
Kβα (x 0.2π)
Kαβ (x 0.2π)
0
-1
0
1

**Supplementary Fig. 9. Blockage of Glucagon Slows Down Islet $Ca^{2+}$ Oscillation**

a) Representative recording of β cell $Ca^{2+}$ signal in *Ins*$^{+/-}$*;GCaMP6f*$^{f/+}$ fast (top panel) and slow (bottom panel) islet. The stimulation used in the experiment is shown above: 3G (10 min), 10G (30 min), 3G + 100 nM MK0893 + 1 uM Ex9 + 7 uM insulin (30 min), 10G + 100 nM MK0893 + 1 uM Ex9 + 7 uM insulin (60 min), 3G (30 min), and 10G (30 min).

b) Representative recordings of normalized WT islet $Ca^{2+}$ signal (loading $Ca^{2+}$ indicator Cal-520 AM) without and with 100 nM MK (MK0893) and 100 nM Ex9 (Exendin (9-39)).

c) Mean $Ca^{2+}$ oscillation periods without (n=12 WT islets from 6 mice) and with (n=19 WT islets from 7 mice) the glucagon blockers (100 nM MK and 100nM Ex9).

d) Mean $Ca^{2+}$ oscillation periods in 31 WT islets from 7 mice and 53 *Ins1-cre;Gcgr*$^{f/f}$ islets from 8 mice. Bars represent mean ± s.d.

e) Mean $Ca^{2+}$ oscillation periods without (n=5 *Ins1-cre;Gcgr*$^{f/f}$ islets from 3 mice) and with (n=20 *Ins1-cre;Gcgr*$^{f/f}$ islets from 4 mice) the glucagon blockers (100nM MK and 100nM Ex9).

f) Schematic of model perturbation for decreasing glucagon downstream target in the phase diagram.

Statistical comparisons in supplementary figs. 8c, 9c and 9e are conducted using two-tailed paired t test. Statistical comparisons in supplementary figs. 1e, 1f, 9d and Table 1 are conducted using two-tailed unpaired t test. See the two-tailed P values in Source Data file. Symbols based on the two-tailed P values are defined as n.s. p>0.1, *p<0.05, **p<0.01, ***p<0.001, ****p<0.0001. Source data in supplementary figures 1e, 2b, 2d, 4a, 4b, 4c, 4d, 5a, 5c, 8b, 8c, 8d, 9c, 9d and 9e are provided as a Source Data file.
