# Supplementary material for: Pancreatic α and β cells are globally phase-locked
Source: Nat Commun. 2022 Jun 28;13:3721. doi: 10.1038/s41467-022-31373-6 (PMC9240067; doi:10.1038/s41467-022-31373-6)
Supplement: Supplementary file 3 — Description of additional supplementary files [file 41467_2022_31373_MOESM3_ESM.pdf]

Description of additional supplementary files

Supplementary Movie 1 : Ca^{2+} fluorescent images of Glu-Cre+; GCaMP6ff/+; Ins2- RCaMP1.07 mouse islet with 3G, 10G stimulation. The video length is 1 hour, 3 s/frame, same islet as in Figs. 2b, 2e, 2f and 3a-e.

Supplementary Movie 2 : Fast Ca^{2+} oscillation images of Glu-Cre+; GCaMP6ff/+; Ins2-RCaMP1.07 mouse islet with 10G stimulation. Noted the globally phaselocked α and β cells, the time resolution is 3 s. Same islet as in Movie 1.

Supplementary Movie 3 : Slow Ca^{2+} oscillation images of Glu-Cre+; GCaMP6ff/+; Ins2- RCaMP1.07 mouse islet with 10G stimulation (see also Figs. 3f up panel and 5c right-up panel). The time resolution is 3 s.

Supplementary Movie 4 : Mixed Ca^{2+} oscillation images of Glu-Cre+; GCaMP6ff/+; Ins2- RCaMP1.07 mouse islet with 10G stimulation (see also Figs. 3f bottom panel and 5D right-bottom panel). The time resolution is 3 s.

Supplementary Movie 5 : Model simulation for four cases as shown in Fig.5c. The red ball represents $\theta\alpha$, and its size is proportional to $1 + \cos(\theta\alpha)$ (insulin concentration). The green ball represents $\theta\beta$, and its size is proportional to G (glucagon concentration).
